# 290 Viral Community-acquired Pneumonia—Antibacterials or Not? A multicenter vignette survey of hospital and emergency medicine physicians

**DOI:** 10.1017/ash.2026.10649

**Published:** 2026-06-23

**Authors:** Paulina Colombo, Justin Hayes, Katherine Ellingson

**Affiliations:** 1 Department of Epidemiology & Biostatistics, Mel & Enid Zuckerman College of Public Health, University of Arizona, Tucson AZ, USA; 2 University of Arizona College of Medicine; 3 University of Arizona, College of Public Health

## Abstract

**Background:**?Residents of long-term care facilities (LTCFs) are particularly vulnerable to infection and colonization with multidrug-resistant organisms (MDROs) due to immunologic vulnerability and prolonged healthcare exposure. Interfacility transfers (IFT) of these residents between LTCFs and acute care hospitals are common. Understanding trends in MDRO admission prevalence among LTCF transfers is important for hospitals considering targeted screening and implementation of protocols to prevent transmission. While the COVID-19 pandemic stalled national progress in MDRO prevention, it is unclear how MDRO admission prevalence trends among LTCFs transfers shifted. We aimed to (1) identify longitudinal trends in MDRO admission prevalence and (2) analyze shifts in these trends relative to the pandemic onset. Methods:?Hospital discharge data from the Healthcare Cost and Utilization Project Arizona State Inpatient Databases were evaluated from Oct 2015–Dec 2023. Acute care admissions for patients over 18 years old with LTCF identified as the admission source were included in the study; 12 epidemiologically important MDROs were identified via ICD-10 codes for each admission. The annual frequency and proportion of patients transferred to hospitals from LTCFs with evidence of MDRO prevalence at admission were described. Longitudinal trends in MDRO admission prevalence among all LTCF-hospital transfers were evaluated using the Cochran-Armitage trend test. Multivariable logistic regression was used to model the odds of MDRO admission prevalence – defined as documented colonization, history, or infection POA – by year, adjusting for age, sex, race, ethnicity, and rurality of patient residence. Results:?Among 14,195 Arizona IFTs from LTCFs to acute care hospitals during the 8.25-year study period, the mean age was 62.6 years, 53.1% were female, and 12.3% contained an ICD-10 diagnosis code indicating MDRO admission prevalence. Of these (n=1,741), 1,341 were documented as MDRO-POA infections, and 256 were documented as carriers or individuals with a known history of an MDRO. From 2015–2023, MDRO admission prevalence among LTCF transfers increased significantly from 6.8% to 14.9% (z=9.3, p<0.0001). Compared to 2016, the odds of MDRO admission prevalence were statistically unchanged for 2017, 2018, and 2019. From 2020-2023 odds increased annually in a dose-response pattern, peaking in 2023 with 58% higher odds compared to 2016 (p<0.001). **Conclusions:** MDRO prevalence among Arizona LTCF-to-hospital transfers more than doubled from 2015–2023. While pre-pandemic rates remained relatively stable, during the post-2020 period, MDRO admission prevalence increased steadily and significantly. These findings underscore the importance of interfacility communication and health record flagging to accurately capture MDRO prevalence upon admission from LTCFs.

